# Riding Toward Danger: A Scoping Review of Burns Associated With Personal Mobility Devices, Including Electric Bikes (E-Bikes) and Electric Scooters (E-Scooters)

**DOI:** 10.1093/jbcr/irae115

**Published:** 2024-06-15

**Authors:** John Warner-Levy, Mohammed Herieka, Zeeshan Sheikh,

**Affiliations:** The Faculty of Biology, Medicine and Health, The University of Manchester, Manchester, M13 9PL, UK; The Adult Burns Centre, Wythenshawe Hospital, Manchester University NHS Foundation Trust, Manchester, M23 9LT, UK; The Adult Burns Centre, Wythenshawe Hospital, Manchester University NHS Foundation Trust, Manchester, M23 9LT, UK

**Keywords:** E-bike, E-scooter, PMD, lithium-ion battery, burn

## Abstract

Burn injuries related to lithium-ion batteries from personal mobility devices, such as electric bikes and electric scooters, have emerged as a global concern. By examining the literature, this study aims to provide an overview of the demographics, patterns, and outcomes of personal mobility device-associated burns. A Singaporean cohort revealed burns resulting predominantly from fires occurring due to the combustion of unattended personal mobility device batteries during charging. In contrast, an Israeli cohort showed a higher total body surface area and highlighted the vulnerability of limbs to burn injuries in such incidents. A Beijing cohort, consisting of pediatric patients indicated potential child safety concerns regarding personal mobility device usage. Finally, a Shanghai cohort demonstrated the potential dangers of personal mobility device battery chargers. The observed differences between those experiencing burn injuries and the broader population of personal mobility device riders in terms of age and gender suggest that younger males may be at higher risk, perhaps due to risky practices such as leaving personal mobility devices unattended while charging. This review emphasizes the need for safety education, adherence to regulations, and responsible consumer choices to mitigate burn injuries. Recommendations include promoting child safety measures, using certified personal mobility devices, and cautious handling of DIY conversion kits. Further large-scale studies are essential to gain comprehensive insights and develop effective safety strategies to protect personal mobility device riders from burn injuries.

## INTRODUCTION

Burn injuries associated with personal mobility devices (PMDs), such as electric bikes (E-bikes) and electric scooters (E-scooters), are becoming a growing global concern.^1^ While PMDs often share general characteristics, there is no standardized definition of what constitutes one. Definitions vary by local law, usually decided by factors such as dimensions, weight, and the presence of an electric motor and wheels. For this study, we broadly defined PMD as “any device powered by an electric motor designed for the transport of one individual.”

Failure of lithium-ion batteries (LIBs) found within PMDs can lead to a phenomenon known as thermal runaway, a process in which excessive heat production generates a progressive positive feedback loop.^2^ The significant thermal energy created has the potential to escape and can cause severe burn injuries. Thermal runaway most often occurs during extended periods of excessive charging and is more likely to happen in LIBs with a reduced capacity for heat dissipation, such as larger batteries and those kept in warmer environments.^2^ Older LIBs are more susceptible to thermal runaway due to the structural alterations and increased internal resistance that come with repeated charging cycles.^3^ Although not unique to PMD batteries, it poses a significant safety hazard, particularly as battery energy density increases with advancing technology.^4^

**Figure 1.**
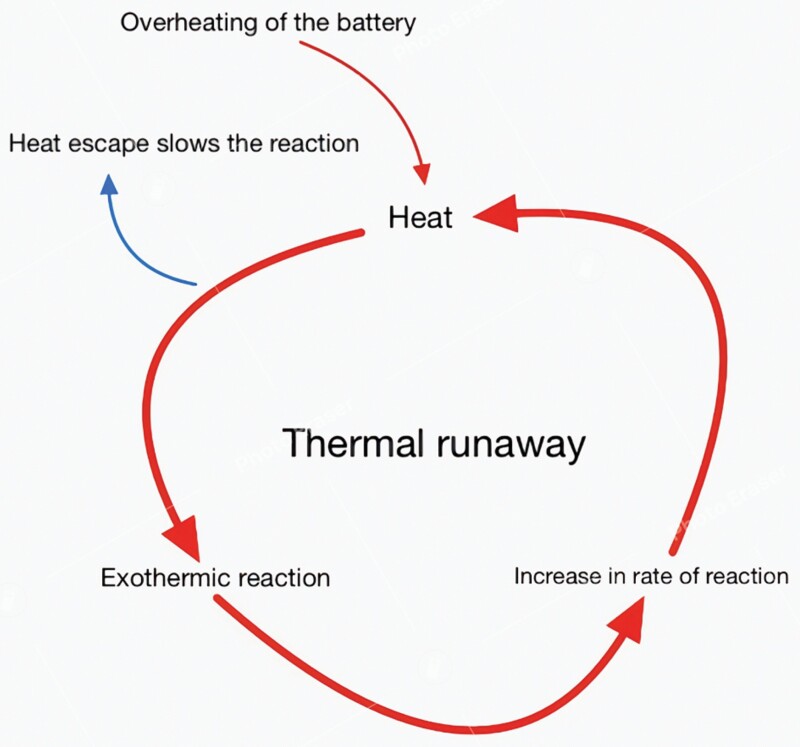
Display of Steps (1) and (2) of Thermal Runaway

As shown in Figure 1, the process of thermal runaway can be divided into three main stages^1^: *(Step 1)* Overheating of the battery can occur secondary to a variety of causes. Of these, internal short-circuit formation, where a path of low resistance forms within the battery and allows a rapid flow of current, appears to be most frequently responsible.^3^ This can occur due to cell defects, such as the internal formation of metallic dendrites, misalignment of internal components, and contamination during assembly.^3^*(Step 2)* The rapidly increasing temperature inside the LIB results in an exothermic reaction, forming a positive feedback loop of ever-increasing levels of heat. This precipitates the emergence of numerous chemical reactions, along with the release of flammable gases, including H_2_, CH_4_, C_2_H2, C_2_H_6_, and CO, and a resultant increase in pressure.^5^ (*Step 3*) When heat levels are sufficient, this can lead to the combustion of flammable gases, resulting in an explosion.

This study aims to provide an overview of the demographics, outcomes, and patterns of burns sustained from PMD-associated injuries.

## MATERIALS AND METHODS

### Protocol and registration

Our protocol was drafted using the Preferred Reporting Items for Systematic Reviews and Meta-analysis Protocols Extension for Scoping Reviews (PRISMA-ScR), which is available to view at https://static1.squarespace.com/static/65b880e13b6ca75573dfe217/t/65b9e60d891cf662fa5f7c13/1706681870986/PRISMA-ScR-Fillable-Checklist_11Sept2019.pdf [accessed 2nd April 2024].

### Eligibility criteria

No restrictions were applied to our literature search regarding study types or language. To be eligible for inclusion, reports must provide instances of burns associated with PMDs. Reports relating to burns associated with electric cars were excluded as, according to our definition, they do not constitute PMDs.

### Information sources

To identify potentially relevant documents, we searched the following bibliographic databases: PubMed, Embase, and Scopus. The search results were exported into EndNote, and duplicates were subsequently removed.

### Search

A review of the literature dated between January 1, 2010 and April 24, 2024 was undertaken with the search terms ((“E-bike*” OR “E-cycle*” OR “E-bicycle*” OR “electric bike*” OR “electric bicycle*” OR “E-scooter*” OR “electric scooter*” OR “E-vehicle*” OR “electric vehicle*” OR “personal mobility device*” OR “PMD”) AND (“burn*” OR “detonation*”)).

### Data items and synthesis of results

Articles were reviewed and grouped by the nature, severity, etiology of burns, patient demographics, clinical management, and trends.

### Critical appraisal of individual sources of evidence

To assess the characteristics, bias, and methodological quality of the included studies, we used the Joanna Briggs Institute Critical Appraisal Tools for use in Case Series and Case Reports, available to view at https://jbi.global/critical-appraisal-tools [accessed 4th April 2024]. For case series, each study was scored on a basis of 10 questions, and for case reports, each study was scored on a basis of eight questions. Our results are attached in Supplementary Table 1. Each study was assessed by two reviewers, and any disagreements were settled by a third reviewer.

## RESULTS

### Selection of sources of evidence

**Figure 2.**
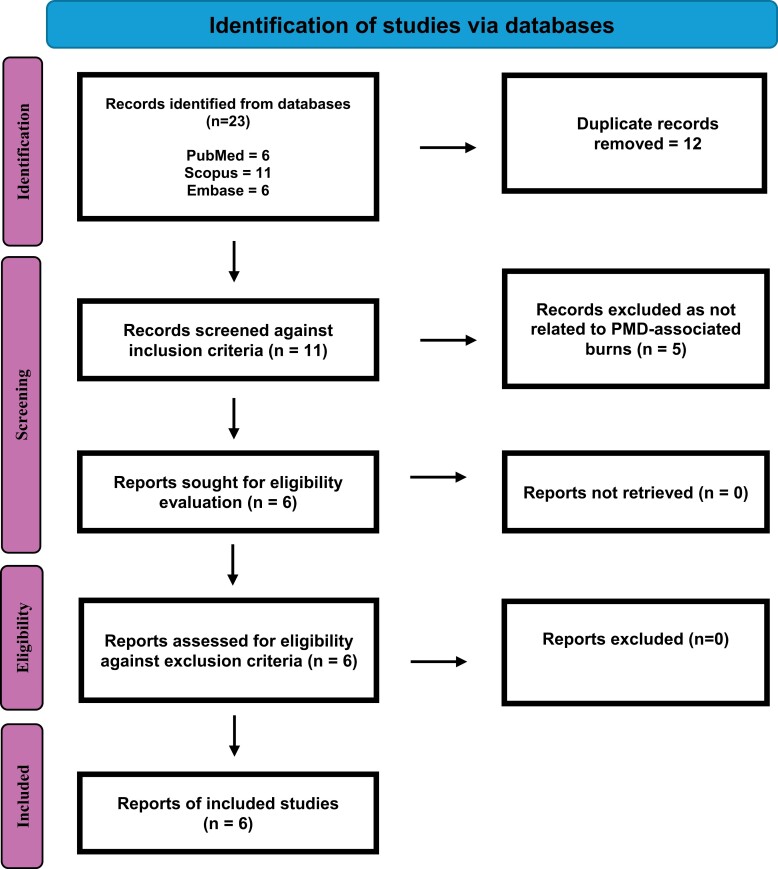
PRISMA Flowchart Showing the Processing of Studies in Our Review

Our literature search yielded 23 results, 12 of which were duplicates, resulting in 11 articles to be screened. Five of these were excluded, as they did not relate specifically to PMD-associated burns. The remaining six papers consisted of two case reports, published in France^6^ and Singapore,^7^ and four case series, published in Singapore,^1^ Israel,^8^ Beijing, China^9^ and Shanghai, and China.^10^ As the Singaporean case report met the criteria for inclusion in the Singaporean case series, it was not included separately in our analysis. Figure 2 contains a PRISMA flowchart showcasing the flow of studies in our review.

### Characteristics and results of sources of evidence

The five eligible reports from our literature review comprised 185 cases of burns associated with E-bikes and E-scooters. However, media reports of other forms of PMD-associated burns exist, including electric skateboards^11^ and electric hoverboards.^12^ PMD-associated fires have also been reported with electric unicycles^13^ and segways.^14^Table 1 provides a summary of literature cases surrounding PMD-associated burns, showcasing data on patient demographics and burn information.

**Table 1.** Demographics and Burn Characteristics of Personal Mobility Device-associated Burns. *Mean value where *n* = 1, **Presumed value

Parameter	Singaporean cohort (*n* = 30)	Israeli cohort (*n* = 9)	Beijing cohort (*n* = 82)	*Shanghai* cohort (*n* = 63)	French case report (*n* = 1)
Sex					
Male	24 (80%)	8 (88.9%)	64 (78%)	44 (70%)	1 (100%)
Female	6 (20%)	9 (10.1%)	18 (22%)	19 (30%)	0
Mean age on admission (years)	26.3 (SD = 19.4)	37.8 (SD = 13.7)	3.5 (SD = 2.5)	38.1 (SD = 2.0)	50*
Burn mechanism					
Battery detonation	30 (100%)	9 (100%)	0	0	1 (100%)
Battery charger detonation	0	0	0	63 (100%)**	0
Short-circuiting of battery	0	0	82 (100%)	0	0
Burn depth					
No cutaneous burns	8 (26.7%)	0	0	Unknown	0
First-degree	9 (30%)	1 (11.1%)	82 (100%)	Unknown	0
Second-degree	9 (30%)	5 (55.6%)	0	Unknown	0
Third-degree	4 (13.3%)	3 (33.3%)	0	Unknown	1 (100%)
Burn location					
Upper limbs	Unknown	7 (77.8%)	77 (93.9%)	Unknown	1 (100%)
Lower limbs	Unknown	8 (88.9%)	0	Unknown	1 (100%)
Face/neck	Unknown	5 (55.6%)	0	Unknown	0
Chest/abdomen/back	Unknown	5 (55.6%)	5 (6.1%)	Unknown	0
Presence of inhalational injury	22 (73.3%)	3 (33.3%)	0**	49 (78%)	0
PMDs involved					
E-bikes	0	Unknown	54 (65.9%)	0	0
E-scooters	90%	Unknown	0	0	1 (100%)
Stand-alone PMD batteries	10%	Unknown	28 (34.1%)	0	0
PMD battery chargers	0	Unknown	0	63 (100%)	
Mean TBSA (%)	14.5 (SD = 22.3)	27.5 (SD = 19.6)	<1 (SD not available)	26.4 (SD = 3.5)	22*
Mean hospital stay (days)	17.7 (SD not available)	34.3 (SD = 5.24)	0	24.0 (SD = 3.0)	40*
Management					
Surgical	12 (40%)	7 (77.8%)	0	42 (67%)	1 (100%)
Conservative	18 (60%)	2 (22.2%)	82 (100%)	21 (33%)	0
Mortality rate	3 (10%)	0	0	7 (11%)	0

### Synthesis of results and insights

#### French case report

The authors reported this to be the first documented E-scooter-associated burn involving an E-scooter confirmed to meet European standards, serving as a reminder that while purchasing PMDs certified to meet safety standards decreases the likelihood of experiencing burn injury, it does not eliminate the risk, and caution should always be taken.

#### Singaporean, Israeli, Beijing, and Shanghai cohorts

It is worth noting that while the Singaporean and Israeli cohorts encompass burn admissions related to E-bikes and E-scooters; the Beijing cohort focuses solely on pediatric burn admissions related to E-bikes. Finally, the Shanghai cohort highlights how PMD-associated fires occurring in high-density living environments have an increased risk of leading to group burns.

Together, the four cohorts demonstrate the different etiologies of PMD-associated burns. The most frequent burn mechanism encountered included flame burns due to detonation of the PMD battery/battery charger, making up 100% of all PMD-associated burns in the Beijing and Shanghai cohorts. Additional mechanisms involved short-circuiting of the PMD battery due to the pinching or touching of a metal object to the pins of the charging socket of an E-bike/E-bike battery, resulting in flash burns. Across all PMDs, burns involving the detonation of LIBs consistently led to deeper burns, a higher TBSA, and larger mortality rates. Moreover, the distinctive pattern of injuries observed in the Beijing cohort highlights child safety issues regarding PMDs, particularly concerning batteries lacking protective mechanisms.

## DISCUSSION

### Summary of evidence and wider literature

Although no peer-reviewed literature on PMD user demographics exists, surveys regarding E-bike and E-scooter usage have consistently shown respondents to have a male predominance.^15–17^ However, this gender disparity typically does not reach the levels reported in cohorts associated with PMD-associated burns. Consequently, it is unclear if the higher rates of PMD-associated burns seen in males are a result of increased PMD usage, or due to sex-associated risk factors.

DIY conversion kits for PMDs present a potential risk due to reports of malfunctioning leading to fires, with the London Fire Brigade stating that almost 40% of E-bike fires they attend are caused by these kits.^18^ While no formal studies have analyzed their safety, The British National Fire Chiefs Council advises that consumers should ensure kits are purchased from a reputable seller that complies with British standards and that the compatibility of all parts is verified.^19^

Safety certifications typically address E-bikes and other PMDs, such as E-scooters, as distinct entities. In the European Union, EN 15194 provides legal standards of fire safety for E-bikes.^20^ While UL 2849 certification for E-bikes^21^ and UL 2272 certification for E-scooters and other PMDs^22^ are widely adopted safety certifications globally, they have now become mandatory in New York City^23^ and Singapore.^24^ The U.S. National Bicycle Dealers Association have also announced plans for a database listing all certified E-bikes and E-scooters, expanding upon a current list of E-bikes brands available in the US.^25^ In the UK, regulatory guidelines such as the Electrically Assisted Pedal Cycles regulations, have established criteria for E-bikes to be legally classified as regular bicycles.^26^ In China, GB 42296-2022 (July 2023) and GB 42295-2022 (January 2024) were implemented to provide safety standard requirements for E-bike chargers, and E-bikes, respectively.^27^ Globally, the International Organisation for Standardization (ISO) released ISO/TS 4210-10:2020, which details safety and performance requirements for EPACs.^28^

### Limitations

Despite the valuable insights gained from the literature, several limitations warrant consideration. The small sample sizes of existing reports restrict the generalizability of findings, necessitating larger-scale studies to validate and expand on current knowledge. Moreover, the lack of standardized reporting of burn injury data across studies, along with varying ages and etiologies between PMD-associated burn cohorts, hinders direct comparisons and comprehensive analyses.

### Recommendations

Drawing upon insights gleaned from existing literature, we propose several recommendations to mitigate PMD-associated burn injuries. These include adhering to manufacturer-recommended charging times and not leaving PMDs charging unattended, ensuring the purchase of PMDs certified to meet safety standards such as UL 2489, safely storing unplugged PMD batteries out of reach of small children, and either purchasing DIY conversion kits from reputable sellers or having the conversion performed by a qualified professional.

## CONCLUSION

In summary, this report emphasizes gender disparities between overall users of PMDs and patients experiencing PMD-associated burns. This is possibly due to sex-associated risk factors such as increased risk-taking behaviors or a general increase in PMD usage in males. Safety concerns regarding child accessibility to PMD batteries are also raised, along with the importance of consumer awareness regarding the dangers presented by DIY conversion kits. Further exploration of social and economic factors is likely to provide valuable insights for potential interventions to lower the risk of PMD-associated burn injuries. Addressing the growing dangers posed by PMD-associated burn injuries requires collaborative efforts among policymakers, manufacturers, healthcare professionals, and consumers. We hereby advise that future research should focus on larger-scale studies to help validate previous findings and better elucidate the underlying factors contributing to PMD-associated burn injuries.

## SUPPLEMENTARY MATERIAL

Supplementary material is available at *Journal of Burn Care & Research* online.

irae115_suppl_Supplementary_Table
